# Histiocytic sarcoma in renal transplant patients: a literature review

**DOI:** 10.1186/s13256-023-04140-4

**Published:** 2023-10-03

**Authors:** Mahsa Salehi, Shafi Rehman, Miraa Qutab, Ruba Altheeb, Rashmi Prakash, Hamid Reza Jafari

**Affiliations:** 1 Mazandaran University of Medical Sciences, Mazandaran, Iran; 2 Khyber Medical College, Peshawar, Pakistan; 3 Lahore Medical and Dental College, Lahore, Pakistan; 4 Al-Balqa Applied University, Amman, Jordan; 5 Adichunchanagiri Institute of Medical Sciences, Mandya, India; 6 Isfahan University of Medical Sciences, Isfahan, Iran

**Keywords:** Histiocytic sarcoma, Renal transplant, Immune suppression, Lymphoproliferative disorder, Kidney

## Abstract

**Background:**

Histiocytic sarcoma (HS) is defined as neoplasm resembling morphological and immunophenotypic characteristics of mature histiocytes. It is a rare form of lymphoid neoplasms. Despite advances in treatment and diagnosis of histiocytic sarcoma, majority of cases had poor prognosis due to progressive nature of the disease. In the following article, all reported cases of histiocytic sarcoma in renal transplant patients are reviewed.

**Methods:**

In our literature review, all relevant reports were collected electronically by entering the necessary keywords. A Boolean approach using Medical Subject Heading (MeSH) keywords was implemented. After establishing the inclusion/exclusion criteria, article titles and abstracts were evaluated by Systematic Reviews and Meta-Analyses (PRISMA) standards for 2020. All cases of histiocytic sarcoma in renal transplant patients were included.

**Result:**

Based on our inclusion and exclusion criteria 4 case reports were yielded in this review. Two were males and 2 were females with the mean age of 42.25 years. Fever was the most common symptom. Although tumor originated from the native kidney on one patient, the site of the primary tumor was thorax, oropharynx, and transplanted kidney in the rest. Metastasis was detected in all cases. Prednisone was used for all cases. EBV was positive in 2 cases and negative in one of them. Histology was diagnostic and similar in all cases. Immunohistochemistry was done for 3 cases. Although chemotherapy was done for 3 patients, all 4 cases ended in mortality.

**Conclusion:**

Despite the fact that neoplasms are post renal transplant complications, histiocytic sarcoma is a scarce and fatal entity in such patients. Histological and immunohistochemistry tests are the corner stone in diagnosis of histiocytic sarcoma.

## Introduction and background

Histiocytic sarcoma (HS), a rare neoplasm of histiocytic and dendritic cells, is described as tumor cells with morphological and immunophenotypic features of mature histiocytes in WHO classification [1, 2]. It is an extremely rare disease of adulthood, accounting for few cases out of lymphoid neoplasms. Reported cases of HS had a mean age of 46 years old. Not only gender, but also hereditary predictors were not risk factors for HS [3].

Despite the fact that HS can accompany non-Hodgkin’s lymphoma and germ cell tumors, its etiology remains unknown. Majority of HS patients not only suffer from severe coarse of the disease, but also, HS ends in mortality due to poor prognosis [4]. WHO does not classify histiocytic sarcoma as a type of PTLD [2, 5]. Only 4 cases of Histiocytic Sarcoma developed after renal transplantation are available in English Literature [6–9].

Our aim of conducting this systematic review is to elaborate the clinical and pathological features of Histiocytic Sarcoma that arises secondary to renal transplantation.

## Methodology

To investigate cases of histiocytic sarcoma in renal transplanted patients, we used PubMed, Google Scholar, and ScienceDirect. We collected all relevant reports electronically by entering the necessary keywords. The search cutoff date for databases was February 7th, 2023. We implemented a Boolean approach using Medical Subject Heading (MeSH) keywords which were used for all databases. When establishing the inclusion/exclusion criteria listed below, article titles and abstracts were evaluated. In this review, Preferred Reporting Items for Systematic Reviews and Meta-Analyses (PRISMA) standards for 2020 were followed [10]. MeSH keywords searched in PubMed are summarized in (Table 1).

**Table 1:** Mesh keywords used in all search databases

Search	Query
#1	Histiocytic Sarcoma OR Histiocytic Sarcomas OR Sarcoma, Histiocytic OR Sarcomas, Histiocytic OR (“Histiocytic Sarcoma/blood” [Mesh] OR “Histiocytic Sarcoma/classification” [Mesh] OR “Histiocytic Sarcoma/complications” [Mesh] OR “Histiocytic Sarcoma/diagnosis” [Mesh] OR “Histiocytic Sarcoma/diagnostic imaging” [Mesh] OR “Histiocytic Sarcoma/epidemiology” [Mesh] OR “Histiocytic Sarcoma/genetics” [Mesh] OR “Histiocytic Sarcoma/immunology” [Mesh] OR “Histiocytic Sarcoma/microbiology” [Mesh] OR “Histiocytic Sarcoma/mortality” [Mesh] OR “Histiocytic Sarcoma/pathology” [Mesh] OR “Histiocytic Sarcoma/physiopathology” [Mesh] OR “Histiocytic Sarcoma/surgery” [Mesh])
#2	Renal Transplantation OR Renal Transplantations OR Transplantations, Renal OR Transplantation, Renal OR (“Kidney Transplantation/adverse effects” [Mesh] OR “Kidney Transplantation/mortality” [Mesh] OR “Kidney Transplantation/statistics and numerical data” [Mesh])
#3	Search: (#1) AND (#2)

### Inclusion and exclusion criteria

We formulated our research question and broadly searched into the databases. All articles of histiocytic sarcoma were initially included. Meanwhile, our aim was to investigate data of all reported cases of histiocytic sarcoma occurring after kidney transplant. Hence, articles regarding histiocytic sarcoma in renal transplant patients were the focus of our study and the rest were excluded.

## Results

**Fig. 1:**
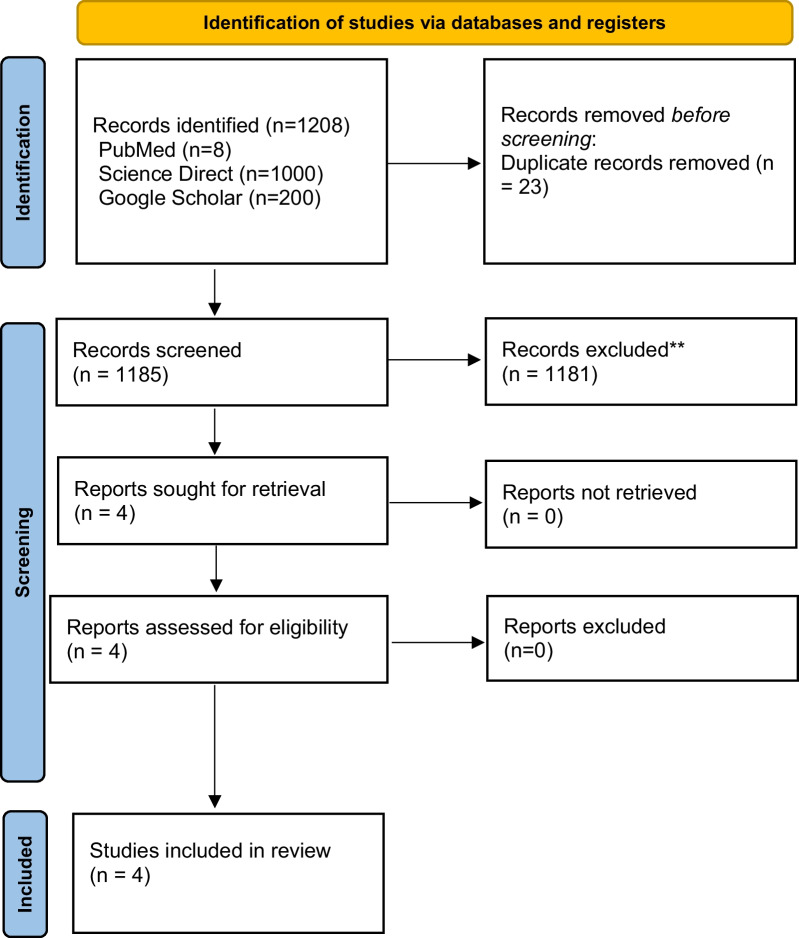
PRISMA of histocyte sarcoma articles

This review includes the aforementioned databases and generated 1208 articles, of which 23 duplicates were removed by Zotero. A total of 1185 records were reviewed, and 1181 were discarded based on the inclusion/exclusion and relevance criteria. The final screening yielded 4 case reports for quality and eligibility evaluation which were all included in this review. A quality assessment tool, the Joanna Briggs Institute (JBI) Critical Appraisal Checklist for Case Reports, was used. The PRISMA flowchart is illustrated in (Fig. 1)*.*

The analysis of the data of 4 case reports are as followed (Table 2):

**Table 2:** Data of histocyte sarcoma in renal transplant patients

Author	Age	Gender	Chief complaints	Primary renal pathology	Native kidney involvement	Site of primary tumor	Metastasis	Immunosuppressive regimen/duration	EBV	Radiological findings	Histopathological findings	Chemotherapy regimen	Prognosis
Pollen *et al.* [6]	57 years	Male	Fever, Fatigue, Loss of appetite and Weight Loss	Chronic Kidney Disease and Hypertensive Nephropathy	Involved	Native Kidney	Yes	Prednisone, Mycophenolate and Cyclosporine for 18 years	Negative	Mass in Liver and Kidney	Histiocytic Sarcoma confirmed via morphology and immunohistochemistry	N/A	Passed away 2 weeks after diagnosis
Ventura Aguiar *et al.* [7]	56 years	Female	Fever and Mass on Right thorax	HCV and CKD of unknown etiology	Uninvolved	Thorax	Yes	Azathioprine and Prednisone for 28 years	EBV serology was positive for IgG but negative for IgM	Multiple thoracic, axillary, pelvic, and abdominal mass	Malignant undifferentiated large cell neoplasm	Thalidomide plus Etoposide but no resolution of disease	Passed away 3 months after diagnosis
Tomlin *et al.* [8]	33 years	Male	Throat pain, Progressive dysphagia and mucoid cough	Glomerulonephritis	Uninvolved	Oropharynx	Yes	Mycophenolate, tacrolimus, and prednisone with no mention of duration	N/A	Multiple supraglottic and tonsillar masses with involvement of cervical lymph nodes, subcutaneous nodules in hip and thigh	Atypical histiocytes with immunohistochemistry suggestive of Histiocytic Sarcoma	ICE regimen, CLAG-M regimen, 2 cycles of weekly vinblastine then myeloablative cyclophosphamide/TBI allogenenic hematopoietic cell transplant from a matched sibling donor	Resolution of Histiocytic Sarcoma but Passed away 9 months after diagnosis due to bacterial pneumonia
Kramer *et al.* [9]	23 years	Female	Paroxysmal paranesthesia and paresis of the right extremities	Congenital anomalies, chronic pyelonephritis and recurrent UTIs lead to renal insufficiency	Uninvolved	Transplanted Kidney	Yes	Azathioprine and Prednisone for a year	EBV serology was positive for IgG but negative for IgM	Multiple supra- and infratentorial brain masses, ovary, and leg	Histiocytic sarcoma confirmed due to large cells with characteristic markers that correspond to the normal cells of the mononuclear phagocytic system (MPS) occurring extra-nodal. it was called HS	Cyclophosphamide, Doxorubicin, Vincristine, and Prednisone	Chemotherapy didn’t influence the progression rate of the tumor and the patient died 4 weeks after the diagnosis

### Demographics

In our review, there were 2 males and 2 females as per gender distribution. The mean age of patients was 42.25 years in a range of 23–57 years. Data regarding ethnicity and gender were insignificant.

### Clinical presentation

Fever was the most common symptom. However, progressive dysphagia, paresthesia, and paresis of the right extremities were mentioned as well in the rest. Moreover, weight loss was more prominent one of the reported patients. The primary renal pathologies were chronic kidney disease in 2 cases, glomerulonephritis in 1 case, and pyelonephritis with recurrent UTIs in 1 case. Also, histiocytic sarcoma originated from the native kidneys in a patient. In the rest, the primary site of the primary tumor was thorax, oropharynx, and transplanted kidney. Furthermore, metastasis was reported in all cases.

### Immunosuppressive regimen and duration

All patients received prednisone followed simultaneously by Azathioprine in 2 cases and Mycophenolate in the rest. Additionally, Cyclosporine and Tacrolimus were used in one of the cases. Duration of the immunosuppressants consumption ranged between 1 and 28 years.

### Virological profile for EBV

Out of 3 cases tested for EBV infection, EBV serology (IgG) was positive in 2 cases and it was negative in another one.

### Histopathology

**Fig. 2:**
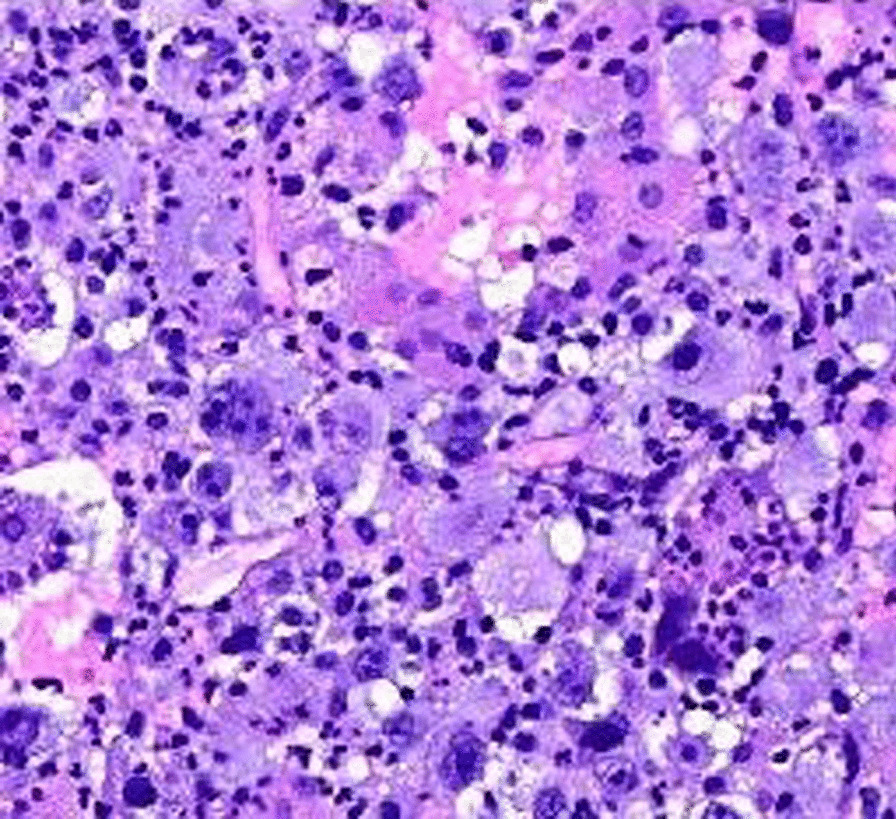
Histiocytic Sarcoma H&E [7]

**Fig. 3:**
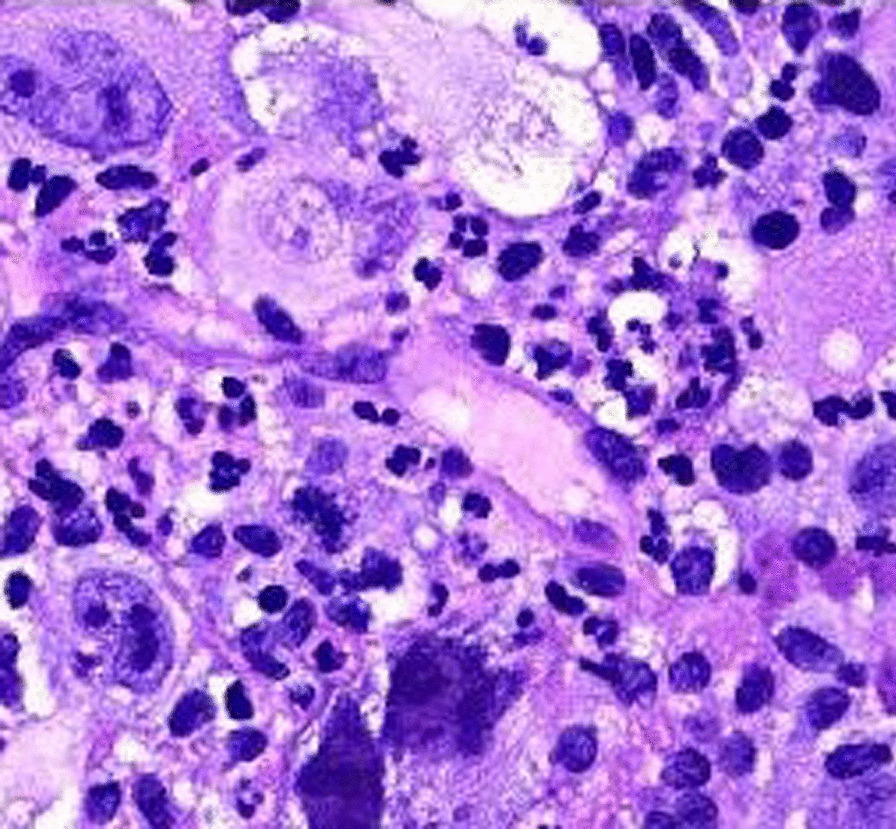
Histiocytic Sarcoma H&E [7]

Histopathological study was discussed in all 4 cases which revealed collection of malignant undifferentiated large cell tumors resembling atypical histiocytes features. In addition, marked polymorphism and mitosis were frequently seen. Moreover, tumor cells were non cohesive and their infiltration into the surrounding stroma was prominent. Hence, morphology along with immunohistochemistry (IHC) was used in the path of reaching a precise diagnose of Histiocytic Sarcoma (Figures 2, 3).

### Immunohistochemistry

**Fig. 4:**
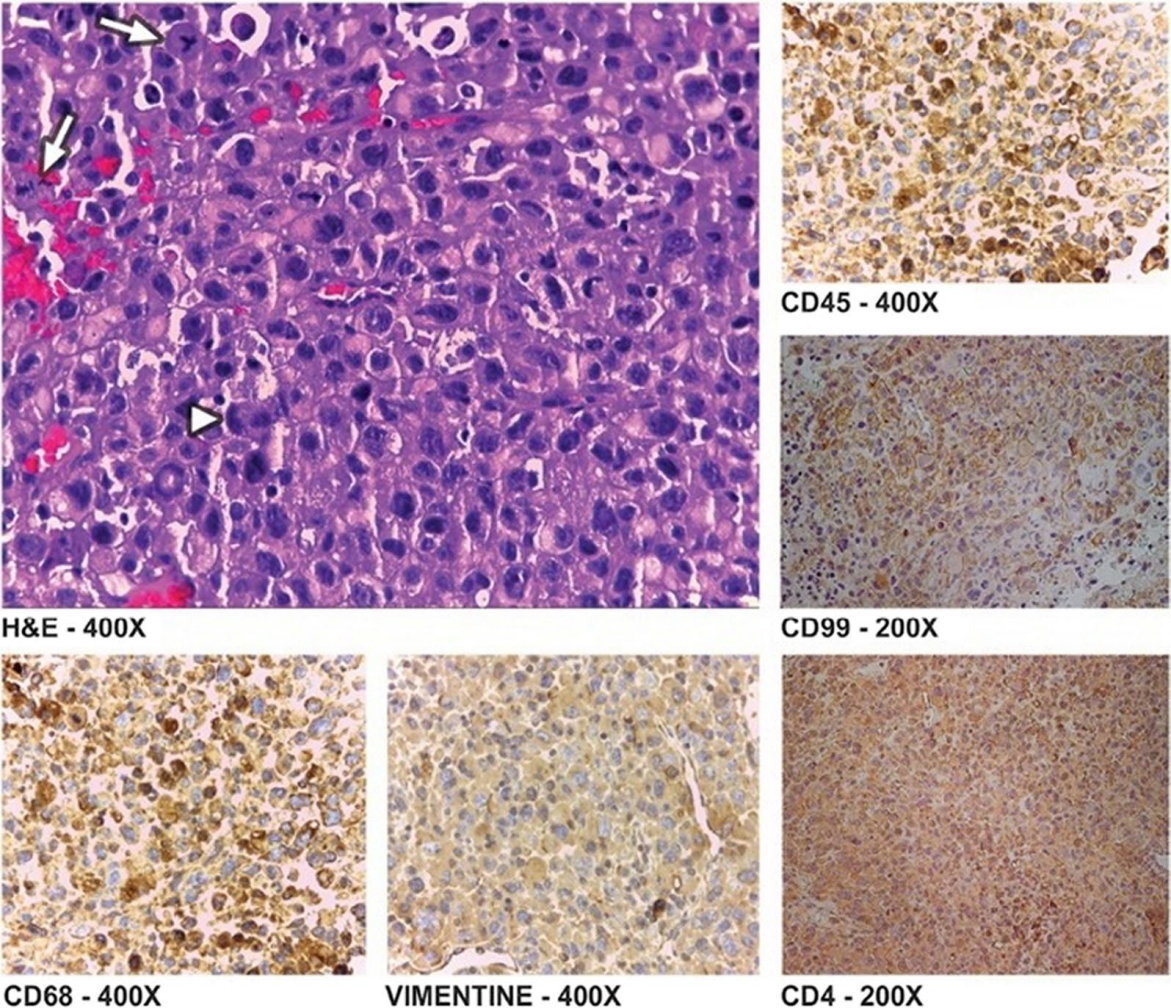
H&E and IHC panel of a histiocytic sarcoma of a transplanted kidney. Focal emperipolesis are marked by (arrowhead) and (arrows) point out frequent mitosis [6]

Immunohistochemistry panel was tested in 3 cases. Regarding IHC, CD 68 in all 3, CD 4 in 1 case, CD 99 in 1 case was positive. Nonetheless, CD 99 was negative in 1 case and S100 was positive in 1 case and negative in another one. Moreover, HAM-56 and Vimentin were positive in 1 case. Also, Lysozyme was positive in 2 cases (Table 3 and Figure 4).

**Table 3:** IHC panel of histocyte sarcoma in renal transplant patients

Markers	CD 4	CD 68	CD 99	S100	HAM 56	Vimentin	Lysozyme
Case No 1	–	+ ve	− ve	− ve	+ ve	–	+ ve
Case No 2	+ ve	+ ve	+ ve	–	–	+ ve	–
Case No 3	–	+ ve	–	+ ve	–	–	+ ve
Case No 4	–	–	–	–	–	–	− ve

### Chemotherapy regimen

Three cases went under Chemotherapy regimen which its regimen consisted of Thalidomide plus Etoposide, ICE regimen, CLAG-M regimen, Cyclophosphamide, Doxorubicin, Vincristine, Vinblastine, and Prednisone.

### Outcome/follow-up

The tumor resolved after initiating the chemotherapy regimen in one of the patients despite the fact that the tumor remained unresolved in the other 2 patients. In one patient, there was no mention of chemotherapy regimen. Their follow-up ended in mortality in all cases which occurred 4 weeks to 9 months after the diagnosis of histiocytic sarcoma.

## Discussion

### Post-transplant lymphoproliferative disorder (PTLD)

Post-transplant lymphoproliferative disorder (PTLD) is a commonly known complication in allograft recipients who were treated by immunosuppressive medications. It has been reported in 1–2% of renal transplant patients [11]. PTLD is a chronic complication of transplantation and consists of hyperplastic-appearing lesions to frank non-Hodgkin’s lymphoma, multiple myeloma histology, and T-cell lymphomas [6].

The most common tumors that develop after renal transplantation are skin tumors, malignant lymphomas, Kaposi’s sarcoma, and cervical carcinoma [12]. The majority of malignant lymphomas are B-cell lymphoproliferative disorders that occur more frequently in transplant recipients than general population in the same age category [13]. According to half of the reported cases, the central nervous system is involved, compared to less than 1% involvement of CNS in lymphoma patients in general. In 30% of such patients, the transplanted organs are involved as well [14]. Moreover, transplant recipients are at high risk of being infected with viral diseases predominantly caused by members of the Herpetoviridae and Papovaviridae. It may not be just a coincidence that the common malignancies after transplantation such as skin tumors, malignant lymphomas, Kaposi’s sarcoma, and cervical carcinoma are in correlation with such viruses such as papilloma/polyoma-like agents, Epstein-Barr virus (EBV), cytomegalovirus and herpes simplex virus, respectively [15, 16].

### Histiocytic sarcoma definition

Histiocytic sarcoma is the proliferation of malignant cells demonstrating morphological and immunohistochemical features of mature histiocytes. Histiocytic sarcoma incident is unknown to due to its scarcity and undefined pathogenesis, nonetheless less than 1% of all hematolymphoid neoplasms is dedicated to HS [2]. Considering the age, the age groups of 0–29 years and 50–69 years are mostly affected by HS [1, 2, 5]. Generally, midline germ cell tumors, preexisting lymphoma/leukemia, viral infection, and transplantation are associated with HS [17]. There are reports of HS diagnosed in preexisting hematopoietic malignancies, mostly stem cell transplant cases, which point toward the trans-differentiation of B-cell neoplasms to HS. On a molecular level, some pathways were hypothesized, in spite of not being thoroughly proved.

HS can involve both nodal and extra-nodal sites of the organs such as gastrointestinal tract, spleen, soft tissue, and skin [3, 18, 19]. Nevertheless, solid organ involvement of HS is not as common. Four cases of HS in postrenal transplant individuals have been reported in English literature. One of the four cases was diagnosed within a year of transplantation [9] and in the other 3 cases HS occurred 10 years after renal transplantation [7, 8]. In all the cases, HS demonstrated advanced stage of multifocal mass lesions with similar morphologic features ranging from collection of atypical histiocytes to malignant undifferentiated large cell tumor [6–9].

### Diagnosis of HS

The diagnosis of HS is based on morphology. A vast immunophenotypic analysis is established to verify histiocytic lineage and exclude poorly differentiated large cell malignancies [17]. The main differential diagnosis are Langerhans cell histiocytosis, dendritic cell sarcoma, diffuse large B-cell lymphoma, anaplastic large T-cell lymphoma, myeloid sarcoma/AML, undifferentiated carcinoma, and malignant melanoma [1, 3, 17]. The consistent similar morphologic findings described in literature could assist a pathologist in diagnosing HS at the time of first encounter either on cytology or on needle core biopsy, mentioned by Pollen *et al.* [6]. Moreover, the morphologic features can aid in making the distinction from reactive histiocytic proliferations. Such tumor is characterized by mainly dissociated single, large neoplastic cells, large pleomorphic nuclei, prominent nucleoli, and abundant eosinophilic to vacuolated cytoplasm. Although hemophagocytosis is classically described in HS, more recent case series revealed that it was only a feature of a subset of cases [3, 17].

### Immunohistochemistry of HS

Immunohistochemistry plays a major role in detection of clonal histiocytic proliferation due to inconsiderable findings on electron microscopy and lack of universal genetic markers [3]. A strict criterion is that the neoplastic cells must express at least two specific macrophage-associated antigens. Typically, lack of B-cell and T-cell markers and Langerhans cell (CD1a, langerin/CD207), follicular dendritic cell (CD21, CD23, CD35, and CAN.42), epithelial (pancytokeratin, EMA), melanocytic (HMB-45, Melan A), and myeloid cell (CD13, CD33, myeloperoxidase) markers have been proposed to diagnose rare cases of bona fide histiocytic tumor [1, 3, 17]. Moreover, potential pitfalls included occasional expression of CD45 and CD4. Langerhans cell markers CD1a, S100, and the follicular dendritic cell marker podoplanin (D2-40) were expressed by a subset of HS [3, 17]. On the other hand, CD163, a hemoglobin scavenger receptor, has been recognized as a new macrophage-related differentiation marker, with higher specificity for histiocytic origin in comparison to other histiocytic markers such as CD68 [20]. More recently, T-cell immunoglobulin mucin 3 and T-cell immunoglobulin mucin 4 (TIM-3 and TIM-4) have been used as markers of histiocytic and dendritic neoplasms; however, due to their expression on dendritic cell neoplasms, Langerhans cell histiocytosis, and cases of acute monocytic leukemia, they might not be an ideal marker for confirmation of HS disease [11].

### Immunosuppression and HS

The incidence of malignancies following renal transplant has been speculated to be affected by a number of contributing factors including the carcinogenicity of the anti-rejection agents, suppression of immune surveillance mechanism, chronic antigenic stimulation, and transformation by viruses [6]. Long-term immunosuppression by drugs such as Steroids and Azathioprine can delay gene transcription inhibition ending in mutation of the B- and/or T-cells (translational mutations). Such action can lead to differentiation into histiocytes or macrophages and subsequent proliferation of the mutated monoclonal clone [7, 8]. On one hand, steroids are the main immunosuppression agents in solid organ transplantation which can prevent gene induction through inhibiting the translocation of nuclear factor-κB (NFκB) from cytoplasm to nucleus. Consequently, gene transcription and release of inflammatory cytokines are impaired [21]. Azathioprine, functioning as a major myelocyte suppressant, is a purine analogue derivative of 6-mercaptopurine. Moreover, it integrates into cellular DNA to prohibit gene replication and T-cell activation, consequently [4, 21]. In a case described by Aguiar *et al.*, immunosuppression vintage was over 25 years. Aguiar *et al.* hypothesized that the prolonged gene transcription inhibition due to long term treatment with steroids enhanced the risk of mutations in the B and/or T-cells, possibly causing a differentiation into histiocytes or macrophages. Moreover, azathioprine could enhance the risk of translational mutations and proliferation of a mutated monoclonal clone. Although majority of kidney transplant patients receive similar immunosuppression therapy, the incidence of HS is insignificant [7]. Castro *et al.* performed a study on four cases of HS after treatment of acute lymphoblastic leukemia. Furthermore, trans-differentiation of ALL clone as a subtype of histiocytic malignancies proved that HS could be outcome of such treatment [4]. Also, the correlation between prior non-Hodgkin lymphoma and HS suggests trans-differentiation in genetic analysis [2, 22–24]. Despite a very low incidence of HS in the large number of kidney transplant recipients, the role of prolonged immunosuppression as an etiology in the development of this disease is yet to be queried. Moreover, in our systematic review of case reports, there was no documented PTLD prior to the diagnosis of HS, further challenging such known theory of translational mutation.

Besides immunosuppression given pre-or-post transplant procedure, immunosuppression administered pre-transplant has been also been demonstrated to be a risk factor for PTLD [25]. Defining the exact contribution of specific immunosuppressive drugs could be challenging based on the administering induction therapy and the maintenance dosage. Nevertheless, it is likely that the overall immunosuppressive state (and not a specific immunosuppressive agents) predominates [26]. Introduction of calcineurin inhibitor (CNI) immunosuppression was associated with a significant increase in the incidence of non-Hodgkin lymphoma [27, 28]. Treatment with tacrolimus compared to cyclosporine has been associated with an increased risk of PTLD development in some cases, but not all [3, 18, 19, 29]. In a large population-based cohort study, high doses of azathioprine were associated with increased PTLD risk in solid organ transplant recipients [28]. Whereas, mycophenolate mofetil did not affect the risk of PTLD, possibly, because of its antiproliferative and apoptotic role. Overall, data suggest that the collective immunosuppression dosage had more impact on the risk of PTLD in comparison to type of immunosuppressive agent [26]. However, WHO classification does not include histiocytic sarcoma as a form of PTLD and it is recommended to further probe into it [1].

### EBV infection in HS

In 1985, Kramer *et al.* reported the first EBV associated case of HS, a year after kidney transplant [9]. However, due to novelty in immunohistochemistry of B-cell lineage, the association between EBV and HS has been altered and modified numerously [5, 30]. In fact, some evidence overrule the relationship between EBV infection and high probability of HS [1]. Briefly, PTLD represents a spectrum of abnormal lymphoproliferations. Although Epstein-Barr virus (EBV) and PTLD are strongly related, roughly half of PTLD cases are not correlated with EBV [31, 32]. In EBV-positive PTLD, infected B cells expressed EBV proteins, naming primary latent membrane proteins (LMP1, 2A-B) and EBV nuclear antigens (EBVNA1, 2, 3A-C). In EBV-negative PTLD, “hit-and-run” EBV infection, presence of other infectious agents, and chronic immune response by the allograft are some of the underlying etiologies. In regards to the pathophysiology of EBV positive and -negative PTLD based on genomic analysis, EBV-negative PTLD is undisguisable from sporadic lymphoma in immunocompetent patients as well as resembling mutations in the protein TP53 [31, 32]. Meanwhile, the lympho-genesis may differ between EBV-positive and -negative PTLD [33].

### Treatment of HS

No guidelines or established standard treatments have been developed for HS. Due to misdiagnosis of non-Hodgkin lymphomas as HS, lymphoma directed therapy such as CHOP-like regimens have been used despite its unproven efficacy for histiocytic-directed regimens. Not only outcomes have been poor with multifocal diseases thus far, but also, nearly all patients experienced local or distant recurrence of HS within months of treatment [6]. Cladribine, high dose cytarabine, G-CSF, Mitoxantrone, and allogenic hematopoietic stem cell transplantation caused complete remission in a renal transplant patient suffering from HS. Hence, they demonstrated that histiocyte-directed chemotherapy was more efficient than lymphoma-directed therapy. Nevertheless, one patient died nine months after successful allogenic hematopoietic stem cell transplantation from bacterial pneumonia [8]. Survival from HS depends on the stage and location of the tumor as well as patients’ compliance toward targeted chemotherapy [6].

## Conclusion

Our study reviewed all cases of histiocytic sarcoma in renal transplant patients as well as summarizing the main features of histiocytic sarcoma. Histiocytic sarcoma is a scarce entity in neoplasms following renal transplant. Despite conflicting data, histological and immunohistochemistry play a major role in diagnosis of HS. Nonetheless, further clinical studies are required to provide a universal guideline for treatment and diagnosis of HS.

## Data Availability

Not applicable.
